# Transcanal sound recordings as a screening tool in the clinical management of patients with pulsatile tinnitus: A pilot study of twenty patients with pulsatile tinnitus eligible for digital subtraction angiography

**DOI:** 10.1111/coa.13308

**Published:** 2019-03-18

**Authors:** Sander W. J. Ubbink, Rutger Hofman, Pim van Dijk, Marc van Dijk

**Affiliations:** ^1^ Department of Otorhinolaryngology/Head and Neck Surgery University of Groningen, University Medical Center Groningen Groningen The Netherlands; ^2^ Graduate School of Medical Sciences (Research School Behavioral Cognitive Neuroscience) University of Groningen, University Medical Center Groningen Groningen The Netherlands; ^3^ Department of Neurosurgery University of Groningen, University Medical Center Groningen Groningen The Netherlands

**Keypoints**

- In pulsatile tinnitus, the differential diagnosis includes neurovascular pathology, which can be occult on non‐invasive imaging techniques. Therefore, if a clear diagnosis is lacking, digital subtraction angiography (DSA) is indicated to rule out a potentially hazardous vascular lesion, particularly a dural arteriovenous fistula (dAVF). However, a DSA carries a procedural risk of 1%‐2%.
- In a tertiary care setting, the incidence of a dAVF in pulsatile tinnitus patients lacking a diagnosis after non‐invasive imaging is about 25%‐35%. Therefore, the majority of this group of patients is unnecessarily exposed to the risks of DSA.
- We report on 20 consecutive patients in a tertiary care setting with pulsatile tinnitus who were referred for DSA to rule out neurovascular pathology. We found that the absence of a pulsatile sound detected by transcanal sound recordings, excludes a dAVF (100% sensitivity).
- Consequently, the use of transcanal sound recordings as a screening tool may prevent patients for the unnecessary risks of DSA in the diagnostic work‐up of pulsatile tinnitus.
- Conventional peri‐auricular and neck auscultation with stethoscope is not always sufficient to objectify the presence of a pulsatile tinnitus.

## 1 INTRODUCTION

Tinnitus is a common problem, with a prevalence of about 8% in the general population. A pulsatile tinnitus occurs in less than 10% of tinnitus patients. It has an extensive differential diagnosis. In general, the underlying pathology for a pulsatile tinnitus is found in up to 70% of the cases.^1^ Most frequently, the diagnosis can be made based on non‐invasive imaging techniques (CT or CT‐angiography (CTA); MRI or MR‐angiography (MRA)). However, evaluation of flow dynamics is limited on MRI/MRA and CTA and particularly dural arteriovenous fistulas (dAVFs) are difficult to recognise.

**Figure 1:**
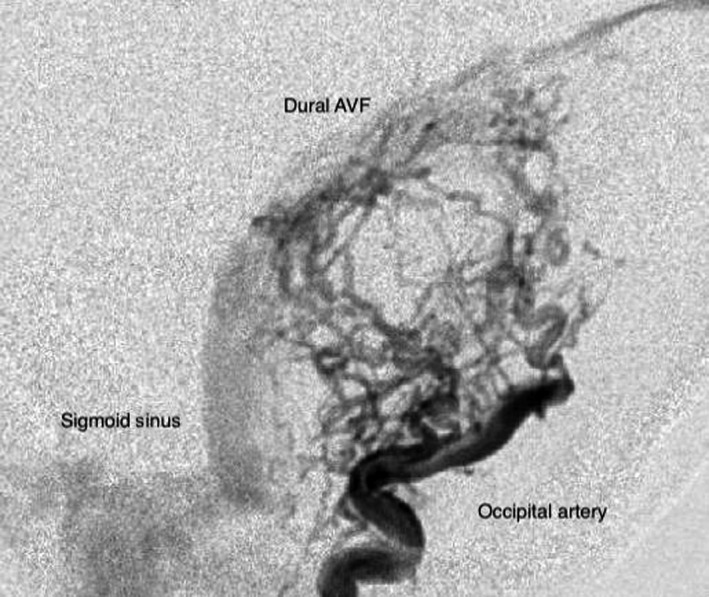
Lateral projection of a digital subtraction angiography examination demonstrating a dural arteriovenous fistula (dAVF)

With an estimated incidence of 0.16 per 100 000 adults per year dAVFs are a rare intracranial vascular malformation.^2^ In more than 10% of the cases of patients with a dAVF, a pulsatile tinnitus is the only initial symptom.^3^ A dAVF with cortical venous involvement (Borden 2 or 3) can, if left untreated, have an aggressive natural disease course with neurological sequelae and even death.^4^ Therefore, patients with pulsatile tinnitus lacking a clear diagnose after non‐invasive imaging should be referred for digital substraction angiography (DSA), which is considered as the gold standard for detection of dAVFs (Figure 1). On the other hand, DSA is an invasive imaging modality, with a procedural risk of 1%‐2% of neurological complications, of which 0.5% are permanent.^5^ In a tertiary care setting, a dAVF was found in 24% of patient with pulsatile tinnitus that underwent a DSA.^6^ As such, the majority of patients with pulsatile tinnitus indicated for a DSA are unnecessarily exposed to these risks. It would be valuable to have a screening tool to narrow indication for a DSA in patients with pulsatile tinnitus.

In some patients, the pulsatile sound is externally detected by a clinician, for example, by auscultation with a stethoscope and sometimes even with bare‐ear listening. Tinnitus is than referred to as objective. The incidence of objective tinnitus in patients with pulsatile tinnitus varies from 6% to 42%.^7^ The incidence of an objective tinnitus in patients diagnosed with dAVF is higher.^8^ Suggesting that an objectified pulsatile tinnitus may be a unique characteristic for patients with dAVF, recently a new method of objectifying pulsatile tinnitus was introduced by measuring sound with a sensitive microphone in the external auditory canal; transcanal sound recordings.^9^ We investigated whether with transcanal sound recordings the indication for DSA in patients with pulsatile tinnitus could be narrowed.

## 2 MATERIALS AND METHODS

### 2.1 Ethical considerations

The Medical Ethics Committee of the UMCG evaluated the research proposal and concluded that the study was not subjected to the Dutch Law on Medical Research with Humans. The board waived the need for patient consent. The study was conducted in accordance with the Declaration of Helsinki and applicable Dutch laws.

### 2.2 Patient selection

The patient cohort for this study consisted of 20 consecutive patients with pulsatile tinnitus that were referred for a DSA to rule out neurovascular pathology. Patients who declined a DSA were excluded from this study. All patients reported a pulse‐synchronous sound that matched their heartbeat. They were all assessed with a physical examination at the ENT department (including otoscope inspection, hearing evaluation, peri‐auricular and neck auscultation with stethoscope), followed by non‐invasive imaging (Table 1). In all patients, the aforementioned examinations did not lead to a definitive diagnosis for the pulsatile tinnitus. Eventually, all patients in this selected cohort underwent a six‐vessel catheter angiography and a transcanal sound recording.

**Table 1:** Clinical image and sound characteristics of the patient cohort

#	Age	Hearing evaluation PTA (dB)	Tinnitus side	Auscultation	CT/CTA	MRI/MRA	DSA (Grade, lateralisation)	Sound measurement
1	29	R:17.5, L:10.0	R	No bruit	Normal	Normal	Normal	Positive
2	66	R:18.8, L:21.3	R	Bruit retro‐auricular	n.a.	Suspicion of dAVF	dAVF (Borden II, R)	Positive
3	66	R:20.0, L:18.8	L	Bruit carotid	n.a.	Suspicion of dAVF	dAVF (Borden I and III, L)	Positive
4	61	R:30.0, L:37.5	R > L	No bruit	Fibrous dysplasia	Suspicion of dAVF	Normal	Negative
5	62	R:26.3, L:21.3	R	No bruit	n.a.	Normal	Normal	Negative
6	71	R:25.0, L:23.8	L	No bruit	normal	Suspicion of dAVF	dAVF (Borden I, R)	Positive
7	69	R:13.8, L:11.3	L	No bruit	Normal	Normal	Normal	Negative
8	54	R:18.8, L:17.5	R	n.a.	n.a.	Normal	Normal	Negative
9	30	R:11.3, L:16.3	L	n.a.	n.a.	Normal	Normal	Negative
10	42	R:10.0, L:10.0	L	Bruit retro‐ auricular	n.a.	Suspicion of dAVF	dAVF (Borden II, L)	Positive
11	53	R:16.3, L:17.5	L > R	No bruit	Normal	Normal	Normal	Negative
12	53	R:11.3, L:25	L	n.a.	Dehiscence sscc	Normal	Cutaneous AVF (‐, L)	Positive
13	29	R:10.0, L:10.0	R	No bruit	Thin tegmen tympani	Normal	Normal	Positive
14	62	R:56.3, L:30.0	L	Bruit retro‐auricular	n.a.	Suspicion of dAVF	dAVF's (L) (Borden I and III)	Positive
15	53	R:28.8, L:20.0	R	No bruit	Normal	Normal	Normal	Negative
16	61	R:27.5, L:27.5	L&R	n.a.	Normal	Normal	Normal	Positive (L&R)
17	37	R:40.3, L:43.8	L	No bruit	Fenestral otosclerosis (L&R)	Normal	Normal	Negative
18	36	R:10.0, L:10.0	R	No bruit	n.a.	Normal	Normal	Negative
19	67	R:18.8, L:18.8	L&R	No bruit	n.a.	Normal	Normal	Negative
20	73	R:32.5, L:33.8	R	Bruit (L&R)	n.a.	Suspicion of dAVF	dAVF (R) (Borden I)	Positive (L&R)

DSA, digital subtraction angiography; dAVF, dural arteriovenous fistula; n.a, not available; PTA, pure tone average 0.5, 1, 2, 4 kHz; sscc, superior semi‐circular canal.

### 2.3 Transcanal sound recordings

The transcanal sound recordings were obtained preceding the DSA. The neurosurgeon who proposed the DSA to these patients was blinded for the result of the recordings. All sound measurements were performed in a sound‐isolated chamber. A microphone (Model ER10B, Etymotic Research Inc, Elk Grove Village, IL, USA) was placed in the external ear canal of the affected ear and a sound registration was made. The signal was amplified and filtered with a bandpass filter (30 Hz‐15 kHz) and sampled at 44.1 kHz. A pulsatile tinnitus was regarded as objective (positive sound measurement) if a pulse‐synchronous sound was present in the recorded signal. If no pulse‐synchronous sound could be detected in the registration, it was regarded as subjective tinnitus.

### 2.4 DSA examination

For the six‐vessel wall catheter DSA, patients were admitted in day‐care. During the procedure the cranial arteries were visualised by using a standard biplane fluoroscopy (ARCADIS Orbic, Siemens, Germany). All DSA examinations were evaluated by a neuroradiologist. If a dAVF was found, it was classified according to the Borden classification.

### 2.5 Statistical analysis

Sensitivity and specificity values were calculated (with 95% confidence intervals; Clopper‐Pearson interval) to analyse the relation of the transcanal sound recording with the outcome of the DSA.

## 3 RESULTS

### 3.1 General characteristics

Between December 2015 and November 2018, 20 patients underwent a DSA to rule out a dAVF and participated in our study. The study included 7 males and 13 females with a mean age of 54 years (SD ± 15). The pulsatile tinnitus was unilateral in 16 out of 20 patients. A CT‐scan was made in 10 of 20 patients. All patients had an MRI/MRA examination. In seven cases MRI/MRA raised a suspicion of a dAVF. None of the patients had non‐invasive time‐resolved imaging (trMRA of 4D‐CTA).

### 3.2 Transcanal sound recordings

A pulsatile sound was detected in the external ear canal of ten patients. In seven patients, DSA revealed an arteriovenous shunt (70%). The angiographic diagnosis was a dAVF in six patients, and an extracranial (subcutaneous) AVF in one patient. In three patients, no abnormalities were found on DSA (false positives). In the remaining ten patients, the sensitive microphone did not detect a pulsatile sound in the ear canal. In none of them, abnormalities were found on DSA.

The sensitivity of sound measurement on the outcome of finding an AVF on DSA was 100% (CI 59%‐100%). Its specificity was 77% (CI 46%‐95%), indicating that in this cohort an objective pulsatile sound yielded no false negatives in detecting an AVF‐shunt. In the same cohort, the sensitivity of detecting an AVF for MRI/MRA and auscultation by stethoscope was calculated 86% and 80%, respectively (Table 2).

**Table 2:** Diagnostic value of examinations on an arteriovenous fistula in PT patients

Examination	% Sensitivity (95% CI)	% Specificity (95% CI)
Transcanal sound recording	100 (59‐100)	77 (46‐95)
MRI/MRA	86 (42‐100)	93 (66‐100)
Auscultation^a^	80 (28‐99)	100 (69‐100)

a In five patients, the outcome for auscultation could not be retrieved.

## 4 DISCUSSION

### 4.1 Synopsis of key findings

Transcanal sound recordings can accurately detect pulsatile sounds in patients complaining of pulsatile tinnitus. In this study, DSA showed no abnormalities if no sound was recorded, while in seven of ten patients with a detectable sound, DSA revealed an AVF. Three patients with an objective pulsatile tinnitus remained without a diagnosis despite non‐invasive imaging and DSA. Our encountered prevalence of dAVF was 35%, comparable with the findings of ‘t Veld et al.^6^

We argue that DSA should be considered if a cause for pulsatile tinnitus is lacking following a conventional diagnostic work‐up, including non‐invasive imaging (CT/CTA and/or MRI/MRA). Particularly in case of a pulse‐synchronous tinnitus, the clinician should rule out a dAVF, a neurovascular entity that may be occult on cross‐sectional imaging. A dAVF regularly presents with a pulsatile tinnitus. If left untreated, a dAVF can have an aggressive natural course with neurological sequelae and even death. On the other hand, DSA is also notorious for its risk on neurological complications. Therefore, it is valuable to have a diagnostic tool to narrow the amount of PT patients eligible for DSA.

### 4.2 Comparisons with other studies

Dural arteriovenous fistulas can be occult on MR imaging. In a tertiary care setting, ‘t Veld et al^6^ found a sensitivity of 75% of MRI/MRA in detecting dAVFs in patients with pulsatile tinnitus. In this study, we found a sensitivity of 86%. This supports that lack of support of an AV‐shunt on MRI/MRA does not preclude the need for a DSA in these patients.

In case a bruit is heard in patients with pulsatile tinnitus, the incidence of vascular pathology is high. It has already been demonstrated that using a conventional stethoscope for auscultation is not sufficient in the detection of objective tinnitus.^10^ In our population of 20 patients eligible for DSA, in 10 patients a bruit could be detected by using transcanal sound recordings. In only four patients, a bruit was objectified by conventional auscultation. These findings demonstrate that simple auscultation is not always sufficient for objectifying the presence of a pulsatile tinnitus.

### 4.3 Strength of the study and limitations

To our knowledge, this is the first study that provides data of a perfect association (100% sensitivity) between objective pulsatile tinnitus, as established by transcanal sound recordings, and a positive DSA. However, the patient numbers are small and further research with a larger patient cohort is necessary to determine the diagnostic accuracy of sound measurements as a diagnostic test.

### 4.4 Clinical applicability of the study

Our study strongly suggests that the absence of a pulsatile sound detected by transcanal sound recordings, excludes the presence of an AVF and therefore the need for a DSA. In contrast, the presence of a pulsatile sound provides a strong motivation to perform a DSA. As such, transcanal sound recordings may prevent patients with pulsatile tinnitus the risks of an unnecessary DSA.

## CONFLICT OF INTEREST

The authors declare no conflict of interest in connection with this article.
